# Supplementary material for: Breast cancer classification based on the integration of diagnostic algorithms for calcifications and masses using a mixture of experts
Source: PLoS One. 2025 Sep 4;20(9):e0331017. doi: 10.1371/journal.pone.0331017 (PMC12410704; doi:10.1371/journal.pone.0331017)
Supplement: Attachment — Submitted filename: comments.docx [file pone.0331017.s001.docx]

The article was reviewed under the title " Breast cancer classification based on the integration of diagnostic algorithms for calcifications and masses using a mixture of experts".

Overall, the focus of the study is good. However, I would like to offer several recommendations that authors may find useful in the process of revising their manuscript:

1- Given the importance of the topic, it is necessary to include a review of previous studies in the article.
2- The phrase " in the supplementary analysis of each radiological finding, statistical testing was not applied, as the purpose was not formal comparison but rather descriptive exploration of model behavior " is used in the text of the article. Please provide a more detailed description of this phrase.
3- Abbreviations in the article are given in a paragraph titled "Abbreviations". It is better to introduce them in parentheses in the text of the article for the first time, and then only use the abbreviations.
4- Considering the results presented for the evaluation parameters, it is better to compare these results with the results presented by other researchers in other articles.
5- The conclusion section is very brief and needs to be rewritten.
